# A review: Aeration efficiency of hydraulic structures in diffusing DO in water

**DOI:** 10.1016/j.mex.2023.102092

**Published:** 2023-03-10

**Authors:** Diksha Puri, Parveen Sihag, M.S. Thakur

**Affiliations:** 1 School of Environmental Sciences, Shoolini University, Solan, Himachal Pradesh 173229, India; 2 Department of Civil Engineering, Chandigarh University, Punjab 140301, India; 3 Professor, Department of Civil Engineering, Shoolini University, Solan, Himachal Pradesh 173229, India

**Keywords:** Aeration Efficiency, Dissolved oxygen, Air entrainment, Hydraulic structures, Oxygen transfer rate, Dissolved oxygen (DO) diffusion in water with various systems.

## Abstract

This paper contemplates the review of aeration efficiency with commonly used different aeration systems such as Venturi flumes, Weirs, Conduits, Stepped channels, In Venturi Aeration, the SAE value grows fast with the number of air holes. In Weir Aeration, it was found that among all the different labyrinth weir structure, triangular notch weirs are known for the optimum results for air entrainment. The ANN model was developed with parameters discharge (Q) and tail water depth (T_w_) which showed that Q is more influential parameter than T_w_. In conduits structure, it was found that circular high head gated conduits have better aeration performance than other conduits. Aeration efficiency in Stepped channels cascades may range from 30% to 70%. The sensitivity analysis with ANN model showed that discharge (Q) followed by number of steps (N) was the most influential parameter in E_20_. Bubble size was the important parameter to undertake when using bubble diffuser. The oxygen transfer efficiency (OTE) in jet diffusers was predicted developing an ANN model. It was found in sensitivity analysis that the input of ‘velocity’ is highly sensitive to OTE. According to literature, jets can provide OTE in the range of 1.91- 21.53kgO_2_/kW-hr.

## Review Highlights

• Aeration, Oxygen solubility in water, Oxygen transfer rate (OTR) and various aeration systems adopted for dissolved oxygen (DO) in water
• Input parameters in different aeration systems which influence DO in water
• Dissolved oxygen in terms of Aeration efficiency (E_20_), Oxygen solubility in water, Oxygen transfer rate (OTR) in different aeration systems

## Graphical abstract

**Figure:**
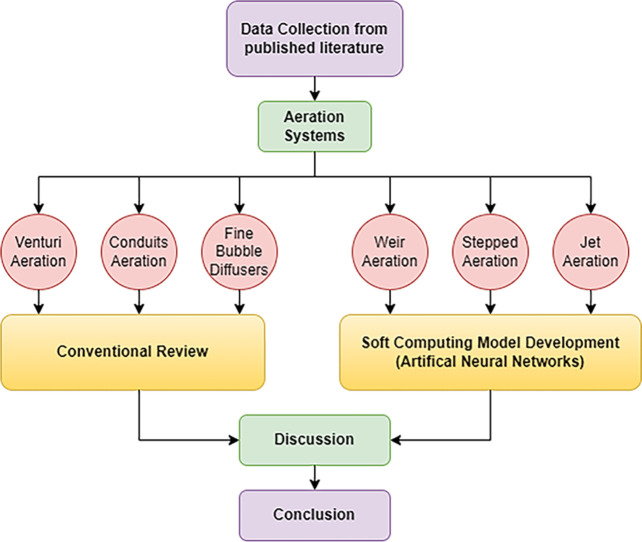

Specifications Table

**Table:** 

Subject area:	Environmental Science, Natural Resource Management
More specific subject area:	Environmental Science
Name of method:	Dissolved oxygen (DO) diffusion in water with various systems.
Keywords:	Aeration Efficiency, dissolved oxygen, air entrainment, hydraulic structures, oxygen transfer rate
Review Question:	1. What are the different systems which can be adopted for dissolution of dissolved oxygen (DO) in water? 2. What are the input parameters in different aeration systems which influence highly for DO in water? 3. To what extent dissolved oxygen (DO) can be achieved in different aeration methods?
Resource availability:	Published research and review articles from Scopus and peer reviewed journals.

## Method Details

Water is a basic resource that is essential to all living entities. In accordance with United Nations Educational, Scientific and Cultural Organization's 2004 projections [64], the amount of water per person is projected to decrease as time passes. Freshwater resources are drastically depleting in the twenty-first century as a result of population increase, urbanisation, industrialization, and the concentration of agricultural operations. Because of phylogenesis and nonpoint sources, nutrient enrichment in fresh systems can result in decreased dissolved oxygen (DO). Anthropogenic activities including the direct release of harsh chemicals into streams, ponds, and rivers have completely destroyed the territorial water ecology [3,14,74,29]. High nutrient levels, poor water transparency, and the development of disease-carrying bacteria and viruses are indicators that these contaminants are becoming more and more prevalent in the water bodies [47,48,50]. The presence poisonous and oxygen depriving cyanobacteria indicates advanced eutrophication, which poses a grave danger to drinking water sources and the environmental manageability of inland water biological systems [40,46]. Oxygen in solution is measured in mg/l or as a percentage of saturation depending on the temperature. When dissolved oxygen levels in water are not saturated, atmospheric oxygen can permeate water bodies as a source of DO. Water plants can also release DO through photosynthesis. The most crucial factor for aquatic bodies is DO. Eutrophication may be to blame for the drop in DO levels in the water column [13,67]. According to Sanchez et al [55], DO is at its best when it is between 5 and 6 mg/L. When the DO extent falls beneath 2 mg/L, the water quality suffers, which can lead to large fish kill.

Since there is considerable air bubble entrainment in the system comprised of hydraulic structures, these are used to increase the oxygen saturation of water [75]. Water only contacts hydraulic structures for a short period of time and provide a boost to dissolved oxygen in river systems. In air bubbles, elevated surface provides the area needed for accelerated oxygen transfer. Air bubbles are formed when air entrains into the flow. In general, hydraulic jumps, plunging jets, and stepped channels act as energy dissipators and self-aerators [38].

The alternative prospects for improving water quality are hydraulic constructions such as stepped spillways, nozzle orifices, or free overflow structures. Stepped spillways, weirs, venturi aerators, and stepped cascades can be used to boost the concentration of dissolved oxygen in a river flow system. Drop-structures, for example baffle blocks, chutes, weirs, and cascades are commonly used in straight flow canals. Gameson [30] was the first to explore weir aeration potential in the river. . Weir was fabricated at the downstream side of the flow for formation of jet. Weir has demerit of high head loss as compared to Parshall and venturi flumes.

## Elements in Aeration efficiency as under:-Aeration, Dissolved Oxygen (DO), Oxygen Transfer Rate (OTR)

### Aeration

The procedure of enhancing the amount of DO in water is known as aeration [32]. Enhancing oxygen transport is crucial to increase energy efficiency since aeration uses the most energy in water resource facilities [1]. Mechanical aeration, or agitating the liquid's surface mechanically, can be employed to introduce oxygen into aeration tanks through diffusion (diffused aeration). An aeration system improves the oxygen transfer required to support a biological process by increasing the air/water contact within a process liquid. Impellers, propellers, or rotors are typically driven by motors during mechanical aeration to provide dissolved oxygen at the liquid surface of aeration tanks. Generally, mechanical aeration systems are divided into categories of: low-speed radial flow, high-speed axial flow, and horizontal rotors with aspiration interfaces. In addition to the equipment used (such as the propeller, rotor, or propeller aspirator), effects of the tank geometry, adjacent walls, and the input power ratio, etc., are also significant. Standard Aeration Efficiency (SAE) is a function of the design of the aeration equipment. Blowers, air pipes, and diffusers are commonly used in diffuse aeration systems, along with instrumentation and controls, to provide oxygen as needed to operate biological processes. Using fine-pore diffusers, more oxygen is transported from the air to the liquid, as a result of providing a greater air-water interface.

Essentially, hydraulic structures involve an artificial waterway that combines water run-off with a man-made structure to store and convey water and mitigate run-off [16].

### Dissolved Oxygen (DO) in water

Gases are generally insoluble in water, especially at high temperatures. Due to the fast movement of gas molecules and their ability to escape from water, they are released fast. This may be due to the lower energy, less oscillation, and closer concentration of water molecules in cooler than in warmer water explaining its greater solubility [12]. The principle of chemical equilibrium can also be used to explain this phenomenon. Oxygen or other gases dissolved in water, release a small amount of heat in the process (Eq. 1).

(1) $$O_{2}\left(g\right)=O_{2}\left(aq\right)+\;heat$$

In warmer water, the process of dissolving oxygen is less efficient because heat accelerates the reaction to the right [28].

In addition, if pressure is low, the gas will be liberated from the water and move freely. The differential in oxygen pressure between the inside and outside of water is what causes oxygen to diffuse into and out of it. When water-pressure is less than the oxygen pressure in air, oxygen diffuses from the atmosphere to the water due to a higher pressure-gradient; Diffusion occurs when the oxygen pressure in water exceeds the oxygen pressure in air. When the oxygen pressures in air and water are equal (in equilibrium), there is no net diffusion of oxygen. While air and water exchange oxygen molecules at equilibrium, the rates of exchange are equal i.e., there is no net diffusion. It is also known as oxygen solubility in water or oxygen saturation concentration.

In consonance with Henry's law, solubility of the gas in water is directly related to its partial pressure in air above the water. The solubility of various gases in water varies, and Henry's law is a constant that identifies the ratio of the gas pressure in air above the water to the gas concentration in water at equilibrium for any given gas [41]. By contrast, atmospheric concentrations are used to express amounts of dissolved gas, while molar concentrations are used to express amounts of gas in the air. In terms of oxygen, Henry's law constant is (Eq. 2):-

(2) $$K_{H}=\frac{\left[O_{2}\left(aq\right)\right]}{P_{O_{2}}\left(g\right)}$$

Where, $K_{H}$ represents the Henry's law constant (M/Atm); $P_{O_{2}}\left(g\right)$ represents the partial pressure of oxygen in air (Atm); $O_{2}\left(aq\right)$ represents dissolved oxygen concentration in water (M).

The solubility of gas will therefore be increased by lowering the temperature and increasing the pressure.

### Oxygen transfer rate (OTR)

Air bubbles per unit volume determine how much oxygen can be transferred through water, as well as their size and distribution along the bubbles. In addition to air and water properties, the geometry of the aeration system and flow parameters are critical Maise [76]. Water and air physical properties such as mass density of air (ρa), mass density of water (ρw), kinematic viscosity of water (υw), and surface tension of water (σw) are important in OTR.

The hypothesis of two-film transfer (of oxygen to water) was put forth by Lewis and Whitman [77] and is based on molecular diffusion in steady state. As a result, the presence of two stagnant equilibrium phases (a liquid and a gas) on either side of the interface may act as a barrier to oxygen molecules passing from the bulk liquid to the bulk gas phase Metcalf and Eddy [78]. When the bulk liquid phase (water) is below the saturation threshold of dissolved oxygen (DO), the oxygen transfer process takes place by molecular diffusion, in which oxygen molecules are moved from gaseous to liquid phases. Oxygen molecules will migrate more from the gaseous phase to the liquid phase when the DO difference between the two phases increases.

The following relationship can be used to calculate the mass transfer rate, r, of oxygen into water (Eq. 3):-

(3) $$r=K_{L}A\left(C_{s}-C\right)$$

Where, $K_{L}\;$= Fluid film coefficient, A = Transfer of oxygen across the interfacial surface, $C_{s}$=Oxygen saturation concentration level in water, C=Water containing oxygen at its present concentration.

While oxygen mass transfer rate depends on the rate of change of oxygen concentration multiplied by the volume of liquid

(4) $$r=\left(\frac{dC}{dt}\right)V$$

In this equation V= Volume of water. As a result, the relationship is given as Eq. (5).

(5) $$\frac{dC}{dt}=\frac{K_{L}A}{V}\left(C_{s}-C\right)$$

Oxygen is believed to transfer from gaseous to liquid phase in three stages:

I Gas-to-liquid oxygen transfer that develops a saturated layer of oxygen at the air-liquid interface (rapid process).
II The process of molecular diffusion transfers oxygen to the surface of a liquid (relatively slow process).
III Diffusion and convection further increase the dissolution of dissolved oxygen in the liquid pool.

Under standard conditions, oxygen transfer coefficient can be determined as shown in Eq. (6).

(6) $${\left(K_{L}A\right)}_{20}={\left(K_{L}A\right)}_{T}\;/\;1.024^{\left(T-20\right)}$$

Where, ${\left(K_{L}A\right)}_{20}$= Transfer efficiency for oxygen at temperature of 20°C; ${\left(K_{L}A\right)}_{T}$= Transfer efficiency for oxygen at temperature of T°C. .

At standard temperature conditions (20°C), the oxygen transfer rate can be calculated as follows (Eq. 7):

(7) $$O_{R}={\left(K_{L}A\right)}_{20}C_{s}^{*}$$

Where, $O_{R}$= rate of oxygen transfer, $C_{s}^{*}$= saturation concentration of dissolved oxygen at standard conditions (20°C).

Actual Oxygen Requirement (AOR) is the amount of oxygen needed to support biological processes. After adjusting for the conditions in the aeration tank, the SOR represents the amount of oxygen that needs to be transferred to meet the AOR. Essentially, Standard Oxygen Transfer Rate (SOTR) is the rate at which the absolute oxygen is transferred into tap water at 20°C with no DO. As a result of field conditions, Actual Oxygen Transfer Rate (AOTR) measures the transfer rate of AOR. The SOTR must be calculated as shown in Eq. (8):

(8) $$SOTR=V\frac{\sum_{i=1}^{n}K_{L}a_{20i}C_{\infty 20i}^{*}}{n}$$

Where, $K_{L}a_{20i}$ = sampling point value of K_L_a corrected at 20°C

$C_{\infty 20i}^{*}$= steady-state dissolved oxygen saturation sampling point value corrected to 20°C.

C*= average spatial dissolved-oxygen saturation concentration in equilibrium

V = volume of the aeration tank

### Element of Soft computing

**Artificial Neural Network:** It is a machine learning technique for numerical forecasting [49], [58]. The theory of an artificial neural network (ANN) had first been developed in the field of biology, where neural networks play a crucial role in the human body. The adjective "neural" refers to a neuron, while "network" refers to a graph-like structure. The term "artificial neural network" describes computer processes based on the basic concept of biological neural networks. ANN is made up of interconnected artificial neurons that are programmed to imitate the qualities of ‘N’ biological neurons. These neurons are cooperating to address a specific challenge. The architecture of ANN includes several user-defined parameters which are set up with neural network-based models (number of hidden layers, learning rate, momentum, and iterations).

### Collecting the information

This review illustrates the different techniques used to achieve DO dissolution in water. This article is based on published articles from Scopus-indexed journals found through a Google Scholar search. The following keyword combinations were used to search for articles on the mentioned search engines: Hydraulic structures + aeration, open channel + oxygen transfer, conduits + efficiency of oxygen transfer, volumetric oxygen transfer + jet diffusers, venturi flumes + efficiency of aeration.

Different systems used, their input parameters to influence DO in water, and the range of DO dissolution achieved are discussed below.

### Venturi Aeration

The term “Venturi” is justified in this instance because the triangular flume is in some ways similar in principle to Venturi tubes or meters. At the air hole of a venturi tube, a venturi system forms a vacuum (air suction) because of a pressure differential. To create a high velocity jet stream in the venturi tube, a pressurized operating fluid (motive) must be introduced into the inlet of the tube. Due to the differential pressure, the increased velocity of the air flowing through the throat section of the venturi tube results in a reduction in pressure in that portion.

In an aeration system using plunging water jet nozzles, Baylar et al. [11] investigated the effect of converging and diverging cone angle and outlet length on flow rate. Using venturi tubes in pipes, [43] investigated how they affected air-water flow ratio (Qa/Qw) and E_20_ during experimental studies where Qa: air entrainment rate (m^3^/s) and Qw: water discharge (m^3^/s). Designed by Ralph L Parshall, the Parshall flume was named after him.

Yadav et al. [72] performed experiments on venturi aeration. It can be stated that At the maximum neck length evaluated in this study, the total combined number of air holes used for air entrainment resulted in a high oxygen transfer coefficient. Various research articles concerned to venturi/Parshall aeration were reviewed as shown in Table 1, in which performance and air entrainment rate is reviewed.

**Table 1:** Venturi Flumes

S. No	Author(s) (year of publication)	Characteristics		Findings
		Geometrical	Others	
				
1	Baylar et al. [11]	J_L_= 0.3m; α: 45°; T_D_=T_L_= 15mm; θ_1_, θ_2_=5° to 20°	R_e_:= 32 × 10^3^ to 224 × 10^3^	Venturi tubes increase their volumetric flow rate of air when R_e_ increases. Air entrainment rate depends on outlet length and θ_2_. Venturi tubes are regarded to be superior in terms of air entrainment when compared to circular nozzles.
2	Emiroglu and Baylar, [25]	N_D_= 19.5mm; J_L_= 0.30 m; Α= 45°;	F_V_= 2.5-15 m/s	Many factors significantly influence the water jet expansion and air entrainment rate on the venturi device Along with the total count, placement, and open/close status of air vents. Venturi devices have greater air entrainment rate than circular nozzles, and greater bubble penetration depth than circular nozzles, thus making them more attractive as a solution to raising DO levels in shallow receiving pools.
3	Ozkan et al. [44] (2006)	θ_1_= 21°; θ_2_=7°; H_D_ = 5.0 mm	F_v_= 1.5 to 4.5 m/s	In this investigation, pond aeration was investigated using high head gated conduit flow systems and two-phase pipe flow systems with venturi tubes. Air vents experience air suction when a high head outlet conduit's gate is partially opened or when there is just a slight difference in pressure between the inlet and outlet sides of the venturi tube. Further, small air bubbles that have been entrained into the water are driven downstream in two phase flow systems. For oxygen to be transferred from air bubbles to water, two-phase flow systems are extremely effective.
4	Yadav et al. [72]	T_L=_ 20-100mm; θ_1_, θ_2_=10^0^,15^0^,20^0^, and 25^0^; N=1-17	F_v_= 1.096m/s	The maximum SOTR and SAE values obtained were 0.0216 kgO_2_/h and 0.611 kgO_2_/kWh, respectively. With increasing T_L_ and θ_1_, θ_2_ the SAE value rapidly increases with N.
5	Dayıoğlu [19]	-	D= 4.1m^3^h^−1^; E= 263.2 kJ/m3 for D and 380 kJ/Kg for OT	The maximum values of SOTR, SAE, and SOTE were 0.206 kgO_2_ h^−1^, 0.681 kgO_2_ kWh^−1^, and 7.32%, respectively.
				

Foot Note Table 1:- Where, R_e_ = Reynolds number; J_L_= Water jet length; α = Angle between water jet discharge and tank; T_D_ = Throat diameter, T_L_= Length of throat; θ_1_ = Converging cone angle; θ_2_ = Diverging cone angle; N_D_ = Diameter of nozzle; F_v_= Flow velocity of water; N= Number of air holes; D= water flow rate; E=specific energy consumption; OT= oxygen transfer; SOTR= Standard oxygen transfer rate; SAE= Standard aeration efficiency; SOTE= Standard oxygen transfer efficiency

### Weir aeration

Gameson [30] introduced the use of weirs to speed up the aeration process in his work on weirs and aeration. When a free water-jet from a weir plummet falls into downstream of flowing water, the air bubbles are entrapped in it resulting in aeration of the water which is a mechanism responsible for higher DO in the water. Four different cross sections for weirs used in the said study are: triangular, rectangular, trapezoidal, and semi-circular. Singh and Kumar [59] studied the role of piano key weir (PKW) in enhancing oxygen concentration in water. Their results showed that with PKW aeration efficiency is enhanced as drop height increases. Another study by Baylar [4] suggests that triangle notch weir has better performnace than other weirs when it comes to enhancing the oxygen concentration in water. Baylar et al. [10] studied the aeration performance of plunging jets from weirs. A critical review of several research articles on weir aeration is shown in Table 2.

**Table 2:** Weirs

S. No	Author(s)	Weir (details)	Discharge (Q)	Drop Height (h)	Findings
1	Watson et al. [69]	Smooth weir Couble faced weir (Rough weir), Cobble-faced chute	0.056 m^3^ /s, 0.11m^3^ /s, and 0.23 m^3^ /s	0.30-0.76m	Rougher the weir, the greater the aeration and lowest with chute. A new model for predicting deficit ratio (r) for cobble-faced weirs was developed, which includes a dimensionless term for tailwater depths.
2	Emin Emiroglu and Baylar [24]	Triangular labyrinth weirs (45°, 90°,135°, and 180°). Weir sill slopes from 0° to 45°	1.0 to 4.0 L/s	0.20 to 1.00 m	At all angles where a weir is present, air entrainment increased as the sill slope did. It has been discovered that angle and sill slope have a substantial impact on the air entrainment rate. At a weir sill slope of 45 degrees, the maximum air entrainment rates were observed.
3	Baylar and Bagatur [5]	Rectangular sharp crested weir, triangular sharp crested weir (45°, 90°, and 135°), trapezoidal sharp crested weir and semi-circular sharp crested weir	1.0 to 4.0 L/s	0.15 and 0.90 m	For the triangular sharp crested weirs, empirical correlations predicting the air entrainment rate and the aeration efficiency (E_20_) were derived. The values derived from the prediction equations and the measured values showed good agreement. While E_20_ declined as discharge increased, air entrainment rate increased at all drop heights in all sharp crested weir types. Additionally, it is noted that E_20_ rises as the angle increases.
4	Kumar et al. [39]	Triangular planform weir	0.0020–0.0125m^3^ /s	0.008m and 0.069m	The efficiency of triangular shaped weirs is superior to that of conventional weirs, is high for low vertex angles, and decreases as the ratio of the head over the crest of the weir and the crest height increases due to interference from downstream water jets.
5	Kim and Walters [37]	Low drop weirs	167 m^3^/h-m, 334 m^3^/h-m, and 502 m^3^/h-m	0.74m, 1.04m, and 1.36m	Tailwater depth should be included for prediction of oxygen transfer at low drop weirs
6	Aras and Berkun [2]	Smooth and stepped weir	0.10-0.40 L/s	-	E_20_ depends on tail water depth. Tailwater depth in a smooth spillway was generally greater than tailwater depth in a stepped spillway. For tail water depths ranging from 0.019 m to 0.040 m, aeration efficiency was found to be 1.376 to 1.897. for smooth weir. Whereas, for stepped weir it was found to be in the range of 1.442 to 1.948 for tail water depth change of 0.016m to 0.037m.
7	Raikar and Kamatagi [51]	Rectangular and triangular weirs and hydraulic jump.	0.5-2 L/s	15-45 cm	Triangular weir aeration effectiveness was found to be 0.1948, while rectangular weir was 0.1012. It demonstrates that triangular weirs perform better in terms of aeration than rectangular weirs. Aeration efficiency for both types of weirs, however, is dependent on drop height. The hydraulic jump produced 0.14285 aeration. For aeration, weirs are more apparent than hydraulic jump.
8	Wormleaton and Tsang [70]	Sharp crested weir Two parallel weirs	1.0-4.3 L/s	500-1,600 mm	Since the overfall jets frequently collide due to the shape of labyrinth weirs, there may be more aeration as a result of the longer sills. Labyrinth weirs with a rectangular planform were the subject of several lab tests. These demonstrated that labyrinth weirs aerated substantially better than an identical straight weir, especially at low drop heights, even when the weir's precise shape was unimportant.
9	Baylar and Emiroglu [6]	Sharp crested weirs: Rectangular, triangular (30°,45°,90° and 135°), trapezoidal and semicircular	5 L/s	0.15 to 0.90 m	Air entrainment rate depends on weir shape. The 30° triangular with two V notches were found to have a higher aeration

In this technique tailwater depth (T_W_) and discharge (Q) influence E_20_ significantly. Therefore, for predicting the E_20_, the soft computing ANN model was applied.

**Figure 1:**
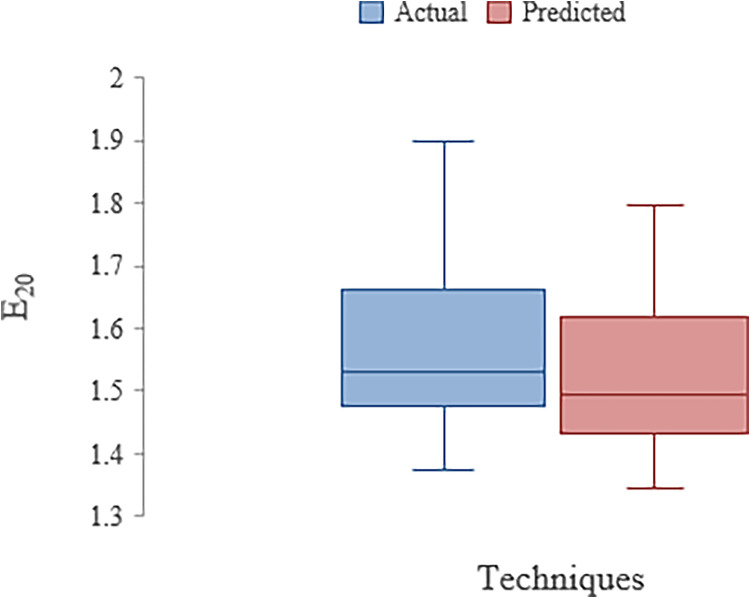
Box Plot with ANN in Weir aeration

To determine the impact of parameters Q and T_w_, the sensitive analysis was carried out which showed that Q is more sensitive than T_w_ in influencing the E_20_ (Table 3). The correlation coefficient (CC) values obtained for the dataset divided in 70:30 percent ratio in training and testing stages are 0.9910 and 0.9864 respectively (Table 4). Table 5 shows the features of weir dataset obtained from literature review. Figure 1 represents box plot for weir aeration in actual and ANN technique. The box plot is the representation skewness of grouped data. It contains information about minimum and maximum values in dataset. In statistics, a quartile is the central determinant of a data set that characterises a division of the observations into four intervals defined by the data values and their comparison with the set of observations. The data set's quartile Q1 is the number that falls between the lowest and highest quartiles. The Quartile Q3 is the number that falls between the data set's median and highest value. The interquartile range (IQR) between Q1 and Q3 is calculated by Q3-Q1.

**Table 3:** Sensitivity Analysis of Weirs (ANN Model)

Variables Combination	Parameter eliminated	CC	MAE	RMSE
				
E_20_= f (Q, T_w_)	-	0.9864	0.0452	0.0501
E_20_= f (T_w_)	Q	0.9174	0.0541	0.0626
E_20_= f (Q)	T_w_	0.9350	0.0596	0.0739

Note Table 3:- Where, E_20_ = Aeration efficiency, Q=discharge, T_w_=tail water depth

**Table 4:** Stat analysis of Weirs in Training and Testing stage (ANN Model)

Description	Training	Testing
		
CC	0.9910	0.9864
MAE	0.0337	0.0452
RMSE	0.0388	0.0501

Note Table 4:- Where, CC= Correlation coefficient, MAE= mean absolute error, RMSE= root mean square error

**Table 5:** Features of weir dataset

	*Discharge*	*Tailwater Depth*	*E_20_*
			
Mean	0.234	0.030613	1.56772
Standard Error	0.012853	0.000789	0.016588
Median	0.2	0.03	1.532
Mode	0.1	0.025	1.509
Standard Deviation	0.111307	0.006834	0.143659
Sample Variance	0.012389	4.67E-05	0.020638
Kurtosis	-1.42477	-1.36128	-0.25489
Skewness	0.328051	-0.08379	0.838031
Range	0.3	0.022	0.521
Minimum	0.1	0.019	1.376
Maximum	0.4	0.041	1.897
Sum	17.55	2.296	117.579
Confidence Level(95.0%)	0.025609	0.001572	0.033053

The minimum values obtained in actual dataset and ANN are 1.376 and 1.345. The maximum values are 1.897 and 1.795 respectively. Q1 values are 1.486 and 1.431 in actual and ANN model. The Q3 values obtained are 1.6525 and 1.617. the IQR values for actual and predicted datasets are 0.139 and 0.186 respectively.

### Conduit Aeration

High pressure is created in sub atmospheric levels when a high-head conduit gate partially opens (Fig 3). The pressure could theoretically be as low as the vapour pressure of water, causing substantial damage due to cavitation. In order to avoid excessive sub atmospheric pressure, an air vent located downstream from the gate connects the conduit to the atmosphere. As a result, the downstream pressure of the gate is kept at a safe level by providing air [79]. Aeration efficiency with conduits structure can be achieved to the greater extent [26,35,56,61,62]. In an experiment conducted by Unsal et al. [66], the researchers investigated the relationship of Froude number with E_20_ (Aeration efficiency in water at 20 degrees Celsius) for the free-surface conduit flow systems. They found that E_20_ increased with increasing Froude number, an effect attributed to increased water velocity at gate locations. Cihat Tuna et al. [18] investigated the aeration efficiency of high-head gated circular conduits and discovered that the Froude number and the ratio of water cross-sectional flow area to conduit cross-sectional area had a significant impact on aeration efficiency. The details of conduit aeration including air entrainment rate, performance of conduits etc. are tabulated in Table 6 and are shown as under.

**Table 6:** Conduits

S. No	Author(s)	Conduit Geometry	Sluice gate Geometry	Froude No.	Findings
1	Ozkan et al. [45]	L-3m; D-68mm	θ=45°;	11.79 and 47.15	Aeration efficiency rises with velocity but falls off at velocities below 10 m/s. Conduit slope and gate opening have no discernible impact on aeration efficiency.
2	Ozkan et al. [44]	D=45mm; H=95mm	θ=45°; φ=5mm to 30mm	-	High head conduits were more aeration efficient in deep ponds. . The SOTR reached to 0.777 KgO_2_/h at velocity of 1.50m^3^.
3	Unsal et al. [65]	L=2m to 6m; W=40mm; H=80mm	θ=45°; φ =1.6cm to 4.8cm	2.726 and 33.798	As Froud no. is increased so as the aeration efficiency. Whereas, neither the conduit length nor the sluice opening has the impact on aeration efficiency. Almost complete oxygen transfers up to saturation was achieved.
4	Ozkan et al. [43]	L=500cm; W=4.5cm; H=9.5cm	θ=45°; φ= 5-20mm		High pressure and air entrainment at the downstream of conduit facilitated aeration efficiency
5	Cihat Tuna et al. [18]	L=2 to 6m	θ=45°	2 and 49	Oxygen can be transferred effectively through high head gated circular conduits. The results revealed that when Fr > 20, the increasing tendency of aeration efficiency decreased.
6	Unsal et al. [66]	L=2m to 6m; W=4cm; H=25cm	θ=45°; φ=1.6cm to 4.8cm	2.73 and 32.42	The outcomes demonstrated the great aeration efficiency of the free-surface conduit flow systems. Nearly full oxygen transfers up to the saturation value was attained at Froude values larger than 15.

Note Table 6; Where, L= Length of conduit; W = Width of conduit; H= Height of conduit; θ= Sluice gate lip

### Stepped Channels

Stepped channels provide an improved hydraulic resistance against flow because they use step laid spillway flooring; when the flow of water passes over the steps, some of the energy dissipates and cavitation is reduced. . Besides high-energy water transfer and flow energy dissipation [80], stepped spillways have low manufacturing costs and high efficiency [82]). Because the crest and downstream portions of a spillway are at various heights, the water velocity increases in the direction of flow [81]. In the stepped channel studies, there are three known flow regimes which take place in stepped channels. A low flow regime (nappe flow regime) results in a wide range of small free falls. Energy is dissipated in three ways: by breaking up jet streams in air, by impacting jets on a step, and possibly by forming hydraulic jumps on the steps. The flow hits each step as a falling jet in the nappe flow regime, causing the energy to be dissipated by jet breakup in air and jet mixing on the step, with or without the formation of a partial hydraulic jump on it [83]). When flow rate increases for a given step geometry, the transitional flow regime may be observed (a step flow pattern) between nappe and skimming flow. The concept of transition flow regime was introduced by Ohtsu and Yasuda [84]. There is a pool of re-circulating water along with a small air cavity, spray water deflection immediately downstream of the stagnant point, and a chaotic appearance associated with the transition flow regime [85].

A coherent stream of water moves over the step in a skimming flow regime; the steps create stable vortices that re-circulate. The transmission of shear stress from the fluid flowing past the steps maintains the vortices [86]. The performance of stepped geometry and various parameters for stepped aeration obtained from various research articles is discussed in Table 7. Six input parameters were obtained from literature review to observe the influence on oxygen transfer with stepped channels. For predicting E_20_ the soft computing ANN model was applied. The sensitivity analysis for six input variables (Table 8) showed that discharge (Q) followed by number of steps (N) is the most influential parameter in E_20_.

**Table 7:** Stepped Channels

S. No	Authors	Geometrical Characteristics	Hydraulics Parameters	Findings
1	Khdhiri et al. [36]	h=0.05-0.10m; l=0.10-0.14m; n=3-10W=0.15-0.3m; H=0.25-0.5m;	Q=0.3-2.5 × 10^−3^m^3^/s h_c_= 0.007-0.03 m	The maximum aeration level reached 70%. In parameter ranges, the standard error for estimating aeration efficiency was found to be less than 17%.
2	Baylar et al. [8]	H=0.05-0.15m; W=0.35m; H=0.45m θ = 30°,40°, and 50°	Q / unit width= 5-50m^2^/s	As step height increases, aeration efficiency generally increases for all chute angles. In comparison to skimming flow, the nappe flow regime offers higher aeration efficiency.
3	Wan et al. [68]	h= 0.1m; n= 9;l=0.25m;W=1m; θ=21.8°	Q= 0.058m^3^/s; h_c_= 0.14m	Turbulent aerated flows accelerate DO diffusion, making it easier to distribute the DO concentrations uniformly in space.
4	Baylar et al. [9]	W=0.30m; H=0.50m; h= 5,10,15cm; θ= 14.48°,18.74°,22.55°,30.40°,and 50°	Q= 16.67 and 166.67L/s	A nappe flow regime produces more aeration than the others
5	Cheng and Chen [17]			CFD model for air-water flow over stepped channel was proposed. Air inception moves downward on the surface of stepped spillways as discharge increases
6	Dermawan et al. [20]	Two set of steps(flat and pooled steps, n=40 and 20); The stepped spillways thickness of 0.01 m and side walls with height of 0.6 m.The slopes of stepped spillway (θ) are 45˚ and 30˚ with number of steps 20 and 40, respectively h=0.025m to 0.05 m-	Discharge per unit width (Q) = 69.13cm^2^/s to 613.38 cm^2^/s and Froude number ranging between 1.12 and 9.91.	Increasing slope of stepped spillways is associated with higher dissolved oxygen levels, especially at Fr* < 2.00, and surface roughness of steps. The increase of dissolved oxygen from the upstream to the downstream of stepped spillway average between 30%-50%.
7	Takahashi et al. [63]	W=1m; n =10; h=0.1m; l=0.25m; θ= 21.8°	Q = 0.017-0.04m^3^/s	When flow conditions were identical on the rough stepped chute and the smooth stepped chute, the position of self-aeration inception was consistently displaced downstream. Local air–water flow measurements also found that the rough step chutes had higher velocities than smooth steps.
8	Moulick et al. [42]	n= 6,8,10,12, and 14; h=0.214,0.25,0.30,0.375, and 0.50m; H= 3.0m; W=0.5m; L= 0.61m	Hydraulic loading rate (q_w_) = 0.001, 0.005, 0.009, 0.013 and 0.0017m^2^/s	The aeration efficiency increases with a dimensionless vale (d_c_/h) and with number of steps.
9	Rathinakumar et al. [52]	h= 0.15 and 0.18m; n= 12 and 10; W=0.60m; H=1.80m	Hydraulic loading rate (q_w_) = 0.005,0.001,0.0015,0.02,0.025,0.03 and 0.035 m^2^/s	Aeration efficiency increases with increase in dimensionless value (dc/h) and increase in number of steps. It was found that aeration efficiency of recycled water was higher
10	Aras and Berkun [2]	n=4; W=7.5cm; H=13.5cm		Aeration happens with the formation of hydraulic jump and water jet.
11	Baylar et al. [7]	h= 5,10,15cm; W=0.30m; H=0.50m	Unit discharge ranged between 16.67 × 10^−3^ m^2^/s and 166.67 × 10^−3^ m^2^/s.	Aeration efficiency in stepped channels is inversely proportional to ratio of critical flow depth to step height.

Note Table 7:- Where, h= Step height; L=Step length; n= Number of steps; W= Channel width; H= Channel height; Q= Discharge; h_c_= Critical water depth, θ= Chute angle

**Table 8:** Sensitivity analysis in Stepped channel aeration with ANN Model

Variables Combination	Parameter eliminated	CC	MAE	RMSE
E_20_= f (Q, α, N, h, l, d_c_/h)	-	0.9848	0.0259	0.0340
E_20_= f (Q, α, N, h, l)	d_c_/h	0.9840	0.0257	0.0324
E_20_= f (Q, N, h, l, d_c_/h)	α	0.9795	0.0290	0.0384
E_20_= f (Q, α, h, l, d_c_/h)	N	0.9238	0.0474	0.0708
E_20_= f (Q, α, N, l, d_c_/h)	h	0.9576	0.0434	0.0572
E_20_= f (Q, α, N, h, d_c_/h)	l	0.9704	0.0363	0.0475
E_20_= f (α, N, h, l, d_c_/h)	Q	0.8723	0.0697	0.0888

Note Table 8:- Where, Q= discharge, α= chute angle, N= number of steps, h= height of steps, l= length of steps, d_c_/h = dimensionless discharge

**Figure 2:**
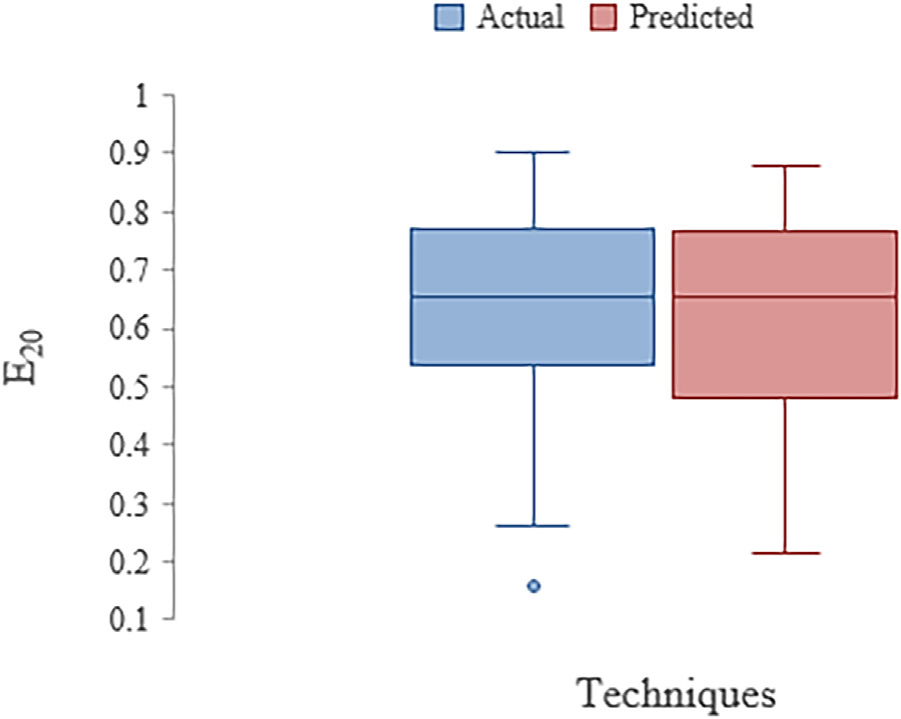
Box plot with ANN in Stepped channel aeration

The CC values obtained in training and testing stages for the dataset bifurcated in 70% and 30% proportion are 0.9891 and 0.9848 respectively (Table 9). Table 10 outlines the features of stepped channel's dataset which were collected from literature review. The result of present analysis is in accordance with Salmasi et al. [54]. They found that chute angle influences nappe regime in stepped spillway. Figure 2 shows box plot for stepped channels for actual and predicted data. It shows minimum and maximum values in dataset. The Q1 obtained from the given box plot for predicted and actual dataset is 0.5425 and 0.4835. The IQR values for same are 0.2275 and 0.2785.

**Table 9:** Stat Indices of Stepped Channel in Training and Testing (ANN Model)

Description	Training	Testing
CC	0.9891	0.9848
MAE	0.0217	0.2590
RMSE	0.0280	0.0340

Note Table 9:- refer to foot note table 4

**Table 10:** Features of stepped channel dataset

	*Q*	α	*N*	*h(m)*	*l(m)*	*dc/h*	*E_20_*
Mean	67.55156	24.62662	20.56954	0.137715	3.37947	0.010513	0.635318
Standard Error	4.51128	1.293062	1.097962	0.008275	0.134109	0.002278	0.014069
Median	50	22.55	16	0.1	3.89	0	0.68
Mode	16.67	0	25	0.05	5	0	0.74
Standard Deviation	55.43554	15.88941	13.49198	0.10168	1.647956	0.027993	0.172887
Sample Variance	3073.099	252.4734	182.0335	0.010339	2.715758	0.000784	0.02989
Kurtosis	-1.03189	-0.91077	0.47085	4.013988	0.282098	7.780124	-0.40753
Skewness	0.490694	0.065948	1.227403	1.958237	-1.17847	2.875505	-0.5691
Range	166.669	50	44	0.45	5	0.1441	0.742
Minimum	0.001	0	6	0.05	0	0	0.16
Maximum	166.67	50	50	0.5	5	0.1441	0.902
Sum	10200.29	3718.62	3106	20.795	510.3	1.5874	95.933
Confidence Level(95.0%)	8.913863	2.554968	2.169468	0.01635	0.264986	0.004501	0.0278

### Fine bubble diffusers

A subsurface diffusion technique called fine-bubble aeration introduces air in the form of tiny bubbles to help or improve the treatment of wastewater [71,73]. The Organization for Economic Cooperation and Development countries now use it the most frequently for wastewater treatment, and it typically has better efficiency per unit of energy used [53]. Due to its straightforward generation and anticipated high dissolution rates in aqueous systems, bubble aeration offers a number of benefits [31,34]. The details related to the bubble aeration are shown in Table 11 as under.

**Table 11:** Fine Bubbles Diffusers

S. No	Authors	Device used	Parameters	Findings
1	Xu et al. [71]	Fine bubble diffusers and impellers	Fr.:15 Hz, 17.5 Hz, 20 Hz, and 22.5 Hz, Q_A_:0.8 m3 /h, 1.2 m3 /h, 1.6 m3 /h, and 2 m3 /h,	As the air flow rate and horizontal liquid velocity increase, so does the oxygen transfer coefficient.
2	Jamnongwong et al [33]	Fine bubble membrane diffusers and large blade slow speed mixers	B_D_(cm), B_V_(cm/s), A(1/m)	The volumetric mass transfer coefficient of liquid film forming apparatus-aeration systems is significantly higher than that of conventional systems.
3	Bunea et al. [15]	Disperse aeration device, fitted with interchangeable perforated plate.	SOTR, SOTE, Re, A, K_La_	A comparison of the KLa performance of various types of aerators is presented.
4	Duchene et al. [23]	38 wastewater plants	SAE, SOTR, SOTE	The small size of the tank implies a low volume/wetted surface ratio in comparison to large open channels, which effectively doubles the mixing power required, with a 7% impact on the SAE.
5	Fayolle et al. [27]	fine bubble diffusers and axial slow speed mixers	A_V_, B_S_, OTC	For various operating conditions, predicted oxygen transfer coefficients are within 5% of experimental results (varying pumping flow rates of the mixers and air flow rates). The actual bubble size must be precisely known in order to estimate the oxygen transfer coefficients with optimism.
6	Zhou et al. [73]	Two sets of identical A^2^/O bioreactors and aeration tank.	K_La_, OTR, OTE	When compared to clean water, the oxygen transfer performance of fine-bubble aerators in the aeration tank decreased dramatically. Under low-aeration conditions, the oxygen transfer coefficient was reduced by more than 50%.

Note Table 11:- Fr.: Working frequency of impeller, Q_A_: Air flow rate, B_D_:Bubble diameter, B_V_: Bubble rising velocity, A: Interfacial area, SAE: Standard aeration efficiency, SOTR: Standard oxygen transfer rate, SOTE: Standard oxygen transfer efficiency, A_V_: Axial liquid velocity, BS: Bubble size, OTC: Oxygen transfer coefficient, K_L_a: Volumetric oxygen transfer coefficient

## Jet Diffusers

A water jet that enters a pool of water at rest after passing through a gas layer (such as the atmosphere) and entrains significant amounts of air is what causes the formation of a submerged two-phase zone beneath the pool free surface. This is known as plunging jet entrainment. This phenomenon is fundamentally caused by the interaction of hydrodynamic and aerodynamic forces between a water jet and the surrounding air. Water jets that plunge downward are employed in a number of industrial and environmental settings. Many industrial processes, particularly those involving air bubble flotation, make use of plunging water jets. In sewage and water treatment facilities, activated sludge is utilised in water jet aeration systems to treat domestic and livestock wastes. Environmental examples include overfall jets from weirs, spillways, and similar hydraulic structures that aerate or purify falling or flowing water by absorbing oxygen from the air. Table 12 shows the literature review on jet diffusers. Discharge (Q), velocity (V) and jet power per unit volume (P/V) are input parameters which are responsible for influencing OTE. To assess the impact of aforesaid parameter, ANN soft computing technique was used to predict the OTE.

**Table 12:** Jet Diffusers

S. No	Authors	Geometrical Characteristics	Parameters	Findings
1	Singh et al. [60]	Circular, square, rectangular and rectangular with rounded edge	K_L_a_(20)_, OTE, A_f_, A_s,_ Q(2.5 × 10^−3^, 2.0 × 10^−3^, 1.6 × 10^−3^, 1.0 × 10^−3^), P/V, P_d_,J_v_	Rectangular with rounded edge plunging jets provided the highest OTE (1.45 times), square (1.68 times), and rectangular (1.36 times) plunging jets at a given kinetic jet power.
2	Deswal and Verma, (2007) [22]	-	K_L_a_(20)_, OTE, n, A_f,_	K_L_a_(20)_ and OTE of m ultiple plunging jets for air/water systems outperformed a single plunging jet significantly.
3	Shukla and Goel, [57]	Rectangular with round edges	K_L_a_(20)_, OTE, J_n_, Q, A_s_	The maximum OTE of 21.53 kgO_2_/kW-hr was obtained for a single nozzle aerator with a discharge of 1.11 l/s;
4	Deswal and Verma [27]	Conical shaped plunging hollow jet	K_L_a_(20)_, OTE, Q, v_j_, t_j_	The results indicate that the conical plunging jet aerator's K_L_a_(20)_ and OTE, are competitive with other types of aeration systems. The OTE ranged between 2.56 – 10.73 kgO_2_/kW-hr
5	Deswal [21]	Single hollow plunging jet	K_L_a_(20)_, OTE, Q, J_t_	K_L_a_(20)_ and OTE increases with discharge. The OTE ranged between 1.91–10.04 kgO_2_/kW-hr

Note Table 12:- K_L_a: Volumetric oxygen transfer, OTE: Oxygen transfer efficiency, A_f_,: Flow area, A_s:_ Jet surface are per unit length, Q: Discharge, P/V: Jet power per unit volume, P_d_: Penetration depth, v_j_: Jet velocity, J_n_ or n: Number of jets, t_j_:_:_ jet thickness

**Figure 3:**
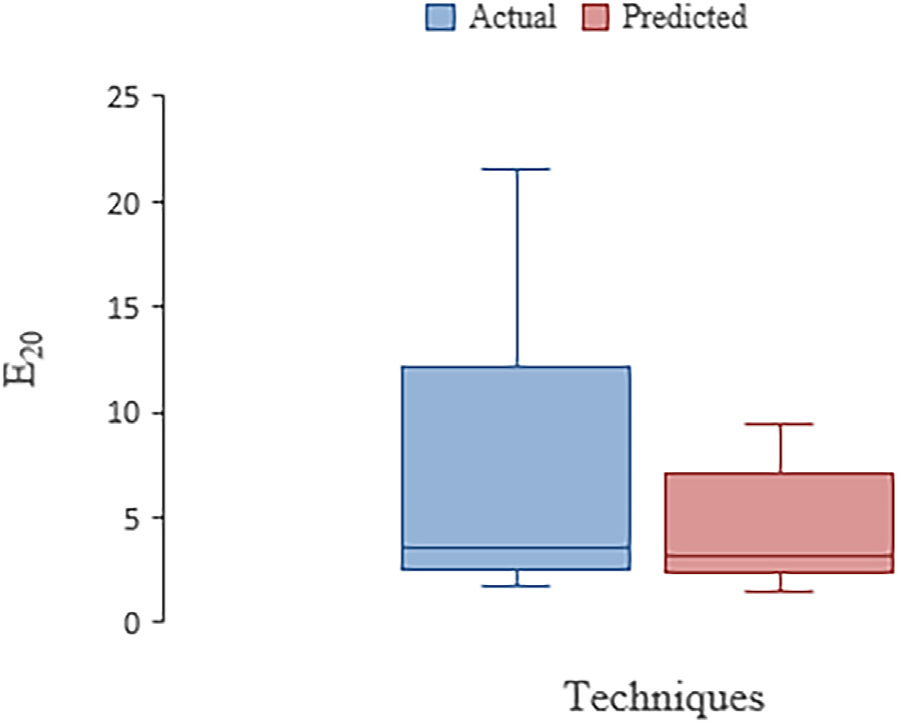
Box plot with ANN in Jet diffuser(s) aeration

The sensitivity analysis performed with aforesaid parameters was carried out which showed that velocity is most influential parameter in OTE (Table 13). Figure 3 shows the box plot for actual and ANN technique. The important parameters in box plot are minimum values for actual and predicted values for E_20_ plotted as 1.73 and 1.465 and the median Q2 values are 3.57 and 3.244 respectively. The maximum value of E_20_ for actual and predicted with ANN model is 21.53 and 9.449 respectively. The IQR values being 7.98 and 2.333 in actual and ANN model. Table 14 shows statistics indices used for evaluating OTE in jet diffusers while Table 15 shows the dataset features obtained for jet diffusers from published literature.

**Table 13:** Sensitivity Analysis of Jet Diffusers in terms of OTE

Variables Combination	Parameter eliminated	CC	MAE	RMSE
OTE_:_ f (Q,V,P/V)	-	0.9143	2.7069	4.5634
OTE_:_ f (V,P/V)	Q	0.9073	2.7621	4.6990
OTE_:_ f (Q, P/V)	V	0.7723	3.5555	6.1114
OTE_:_ f (Q,V)	P/V	0.9129	2.7543	4.6025

**Table 14:** Stat Indices of Jet Diffusers in Training and Testing in terms of OTE

Description	Training	Testing
CC	0.925	0.9143
MAE	0.5955	2.7069
RMSE	0.7173	4.5634

**Table 15:** Features of Jet diffusers dataset

	*Discharge (l/s)*	*Velocity (m/s)*	*Power ÷ Vol*	*OTE*
				
Mean	2.938	8.437	0.315	4.24
Standard Error	0.201399	0.839394	0.068733	0.687702
Median	2.96	7.17	0.13	3.09
Mode	1.11	1.87	0.02	3.15
Standard Deviation	1.273757	5.308793	0.434706	4.349407
Sample Variance	1.622457	28.18328	0.188969	18.91734
Kurtosis	-1.27167	-0.49912	1.998905	7.224343
Skewness	-0.06367	0.785915	1.749628	2.657357
Range	3.58	17.18	1.42	20.48
Minimum	1.11	1.87	0	1.05
Maximum	4.69	19.05	1.42	21.53
Sum	117.52	337.48	12.6	169.6
Count	40	40	40	40
Confidence Level(95.0%)	0.407367	1.697834	0.139026	1.391008
				

## Conclusion

In the present paper different hydraulics structures and their DO dissolution performance have been discussed. After performing the review of the literature and developing an ANN model, it was found in the study that: -

• In Venturi Aeration, in each rising throat length and angle of converging and diverging portions, the SAE value grows fast with the number of air holes.
• In Weir Aeration, it was found that among all the different labyrinth weir structure, triangular notch weirs are known for the optimum results for air entrainment. The ANN model was developed with parameters discharge (Q) and tail water depth (T_w_), which showed that Q is more influential parameter than T_w_.
• In conduits structure, it was found that circular high head gated conduits have better aeration performance than other conduits.
• Aeration efficiency in Stepped channels cascades may range from 30% to 70%. The sensitivity analysis with ANN model showed that discharge (Q) followed by number of steps (N) was the most influential parameter in E_20_.
• In Bubble diffusers, Bubble size is the important parameter to undertake when using bubble diffuser.
• Jet Aeration is a highly reliable method for treating wastewater. The oxygen transfer efficiency (OTE) in jet diffusers was predicted using an ANN model with reliable values of CC as 0.9143, MAE as 2.7069, and RSME as 4.5634 in testing stage. It was found in sensitivity analysis that the input of ‘velocity’ is highly sensitive to OTE. According to literature survey, jets can provide OTE in the range of 1.91- 21.53kgO_2_/kW-hr.

## Limitations of the study

The current study is a review of various structures aimed at increasing DO concentration in water in order to sustain aquatic life and improve wastewater. Due to inadequacy of data in the literature for three aerations systems i.e. venturi, conduits and bubble aeration efficiency, the modelling for prediction of OTE could not be developed. The adequacy of such data can be beneficial in modelling the predictions for OTE.

## Ethics Statements

The data have been taken from the published articles available on the public domain and is duly acknowledged.

## CRediT authorship contribution statement

**Diksha Puri:** Writing – original draft. **Parveen Sihag:** Writing – review & editing. **M.S. Thakur:** Supervision.

## Declaration of Competing Interest

The authors declare that they have no known competing financial interests or personal relationships that could have appeared to influence the work reported in this paper.

## Data Availability

No data was used for the research described in the article.

## Appendix Supplementary materials

Image, application 1
