# Supplementary material for: A review: Aeration efficiency of hydraulic structures in diffusing DO in water
Source: MethodsX. 2023 Mar 10;10:102092. doi: 10.1016/j.mex.2023.102092 (PMC10050780; doi:10.1016/j.mex.2023.102092)
Supplement: Supplementary file 1 [file mmc1.docx]

Table 1: Venturi Flumes

| S. No | Author(s) (year of publication) | Characteristics | | Findings |
| --- | --- | --- | --- | --- |
|  |  | **Geometrical** | **Others** |  |
|  |  |  |  |  |
| 1 | Baylar et al. (2005) | J_L_= 0.3m; α: 45°;  T_D_=T_L_= 15mm;  θ_1_, θ_2_=5° to 20° | R_e_:= 32x10^3^ to 224 x10^3^ | Venturi tubes increase their volumetric flow rate of air when R_e_ increases.  Air entrainment rate depends on outlet length and θ_2_.  Venturi tubes are regarded to be superior in terms of air entrainment when compared to circular nozzles. |
| 2 | Emiroglu and Baylar, (2003) | N_D_= 19.5mm;  J_L_= 0.30 m;  Α= 45°; | F_V_= 2.5-15 m/s | Many factors significantly influence the water jet expansion and air entrainment rate on the venturi device Along with the total count, placement, and open/close status of air vents.  Venturi devices have greater air entrainment rate than circular nozzles, and greater bubble penetration depth than circular nozzles, thus making them more attractive as a solution to raising DO levels in shallow receiving pools. |
| 3 | Ozkan et al. (2006) | θ_1_= 21°;  θ_2_=7°;  H_D_ = 5.0 mm | F_v_= 1.5 to 4.5 m/s | In this investigation, pond aeration was investigated using high head gated conduit flow systems and two-phase pipe flow systems with venturi tubes. Air vents experience air suction when a high head outlet conduit's gate is partially opened or when there is just a slight difference in pressure between the inlet and outlet sides of the venturi tube. Further,  small air bubbles that have been entrained into the water are driven downstream in two phase flow systems.  For oxygen to be transferred from air bubbles to water, two-phase flow systems are extremely effective. |
| 4 | Yadav et al. (2021) | T_L=_ 20-100mm; θ_1_, θ_2_=10^0^,15^0^,20^0^, and 25^0^; N=1-17 | F_v_= 1.096m/s | The maximum SOTR and SAE values obtained were 0.0216 kgO_2_/h and 0.611 kgO_2_/kWh, respectively.  With increasing T_L_ and θ_1_, θ_2_ the SAE value rapidly increases with N. |
| 5 | Dayioğlu, (2022) | - | D= 4.1m^3^h^-1^;  E= 263.2 kJ/m3 for D  and 380 kJ/Kg for OT | The maximum values of SOTR, SAE, and SOTE were 0.206 kgO_2_ h^−1^, 0.681 kgO_2_ kWh^−1^, and 7.32%, respectively. |
|  |  |  |  |  |

Foot Note Table 1:- Where, R_e_ = Reynolds number; J_L_= Water jet length; α = Angle between water jet discharge and tank; T_D_ = Throat diameter, T_L_= Length of throat; θ_1_ = Converging cone angle; θ_2_ = Diverging cone angle; N_D_ = Diameter of nozzle; F_v_= Flow velocity of water; N= Number of air holes; D= water flow rate; E=specific energy consumption; OT= oxygen transfer; SOTR= Standard oxygen transfer rate; SAE= Standard aeration efficiency; SOTE= Standard oxygen transfer efficiency

**Table 2: Weirs**

| S. No | Author(s) | Weir (details) | Discharge (Q) | Drop Height (h) | Findings |
| --- | --- | --- | --- | --- | --- |
| 1 | Watson et al. (1998) | Smooth weir  Couble faced weir (Rough weir),  Cobble-faced chute | 0.056 m^3^ /s, 0.11m^3^ /s, and 0.23 m^3^ /s | 0.30-0.76m | Rougher the weir, the greater the aeration and lowest with chute.  A new model for predicting deficit ratio (r) for cobble-faced weirs was developed, which includes a dimensionless term for tailwater depths. |
| 2 | Emiroglu and Baylar, (2005) | Triangular labyrinth weirs (45°, 90°,135°, and 180°).  Weir sill slopes from 0° to 45° | 1.0 to 4.0 L/s | 0.20 to 1.00 m | At all angles where a weir is present, air entrainment increased as the sill slope did. It has been discovered that angle and sill slope have a substantial impact on the air entrainment rate. At a weir sill slope of 45 degrees, the maximum air entrainment rates were observed. |
| 3 | Baylar and Bagatur, (2006) | Rectangular sharp crested weir, triangular sharp crested weir (45°, 90°, and 135°), trapezoidal sharp crested weir and semi-circular sharp crested weir | 1.0 to 4.0 L/s | 0.15 and 0.90 m | For the triangular sharp crested weirs, empirical correlations predicting the air entrainment rate and the aeration efficiency (E_20_) were derived. The values derived from the prediction equations and the measured values showed good agreement. While E_20_ declined as discharge increased, air entrainment rate increased at all drop heights in all sharp crested weir types. Additionally, it is noted that E_20_ rises as the angle increases. |
| 4 | Kumar et al. (2011) | Triangular planform weir | 0.0020–0.0125m^3^ /s | 0.008m and 0.069m | The efficiency of triangular shaped weirs is superior to that of conventional weirs, is high for low vertex angles, and decreases as the ratio of the head over the crest of the weir and the crest height increases due to interference from downstream water jets. |
| 5 | Kim and Walters, (2001) | Low drop weirs | 167 m^3^/h-m, 334 m^3^/h-m, and 502 m^3^/h-m | 0.74m, 1.04m, and 1.36m | Tailwater depth should be included for prediction of oxygen transfer at low drop weirs |
| 6 | Aras and Barkun, (2012) | Smooth and stepped weir | 0.10-0.40 L/s | - | E_20_ depends on tail water depth. Tailwater depth in a smooth spillway was generally greater than tailwater depth in a stepped spillway. For tail water depths ranging from 0.019 m to 0.040 m, aeration efficiency was found to be 1.376 to 1.897. for smooth weir. Whereas, for stepped weir it was found to be in the range of 1.442 to 1.948 for tail water depth change of 0.016m to 0.037m. |
| 7 | Raikar and Kamatagi, (2015) | Rectangular and triangular weirs and hydraulic jump. | 0.5-2 L/s | 15-45 cm | Triangular weir aeration effectiveness was found to be 0.1948, while rectangular weir was 0.1012. It demonstrates that triangular weirs perform better in terms of aeration than rectangular weirs. Aeration efficiency for both types of weirs, however, is dependent on drop height. The hydraulic jump produced 0.14285 aeration. For aeration, weirs are more apparent than hydraulic jump. |
| 8 | Wormleaton and Tsang, (2000) | Sharp crested weir  Two parallel weirs | 1.0-4.3 L/s | 500-1,600 mm | Since the overfall jets frequently collide due to the shape of labyrinth weirs, there may be more aeration as a result of the longer sills. Labyrinth weirs with a rectangular planform were the subject of several lab tests. These demonstrated that labyrinth weirs aerated substantially better than an identical straight weir, especially at low drop heights, even when the weir's precise shape was unimportant. |
| 9 | Baylar and Emiroglu, (2002) | Sharp crested weirs: Rectangular, triangular (30°,45°,90° and 135°), trapezoidal and semicircular | 5 L/s | 0.15 to 0.90 m | Air entrainment rate depends on weir shape. The 30° triangular with two V notches were found to have a higher aeration |

# Table 3: Sensitivity Analysis of Weirs (ANN Model)

| Variables Combination | Parameter  eliminated | CC | MAE | RMSE |
| --- | --- | --- | --- | --- |
|  |  |  |  |  |
| E_20_= f (Q, T_w_) | - | 0.9864 | 0.0452 | 0.0501 |
| E_20_= f (T_w_) | Q | 0.9174 | 0.0541 | 0.0626 |
| E_20_= f (Q) | T_w_ | 0.9350 | 0.0596 | 0.0739 |

Note Table 3:- Where, E_20_ = Aeration efficiency, Q=discharge, T_w_=tail water depth

# Table 4: Stat analysis of Weirs in Training and Testing stage (ANN Model)

| Description | Training | Testing |
| --- | --- | --- |
|  |  |  |
| CC | 0.9910 | 0.9864 |
| MAE | 0.0337 | 0.0452 |
| RMSE | 0.0388 | 0.0501 |

Note Table 4:- Where, CC= Correlation coefficient, MAE= mean absolute error, RMSE= root mean square error

**Table 5: Features of weir dataset**

|  | *Discharge* | *Tailwater Depth* | *E_20_* |
| --- | --- | --- | --- |
|  |  |  |  |
| Mean | 0.234 | 0.030613 | 1.56772 |
| Standard Error | 0.012853 | 0.000789 | 0.016588 |
| Median | 0.2 | 0.03 | 1.532 |
| Mode | 0.1 | 0.025 | 1.509 |
| Standard Deviation | 0.111307 | 0.006834 | 0.143659 |
| Sample Variance | 0.012389 | 4.67E-05 | 0.020638 |
| Kurtosis | -1.42477 | -1.36128 | -0.25489 |
| Skewness | 0.328051 | -0.08379 | 0.838031 |
| Range | 0.3 | 0.022 | 0.521 |
| Minimum | 0.1 | 0.019 | 1.376 |
| Maximum | 0.4 | 0.041 | 1.897 |
| Sum | 17.55 | 2.296 | 117.579 |
| Confidence Level(95.0%) | 0.025609 | 0.001572 | 0.033053 |

**Table 6: Conduits**

| S. No | Author(s) | Conduit Geometry | Sluice gate Geometry | Froude No. | Findings |
| --- | --- | --- | --- | --- | --- |
| 1 | Ozkan et al. (2014) | L-3m; D-68mm | θ=45°; | 11.79 and 47.15 | Aeration efficiency rises with velocity but falls off at velocities below 10 m/s. Conduit slope and gate opening have no discernible impact on aeration efficiency. |
| 2 | Ozkan et al. (2006) | D=45mm;  H=95mm | θ=45°; φ=5mm to 30mm | - | High head conduits were more aeration efficient in deep ponds. . The SOTR reached to 0.777 KgO_2_/h at velocity of 1.50m^3^. |
| 3 | Unsal et al. (2008) | L=2m to 6m; W=40mm; H=80mm | θ=45°;  φ =1.6cm to 4.8cm | 2.726 and 33.798 | As Froud no. is increased so as the aeration efficiency. Whereas, neither the conduit length nor the sluice opening have the impact on aeration efficiency. Almost complete oxygen transfer up to saturation was achieved. |
| 4 | Ozkan et al. (2006) | L=500cm; W=4.5cm; H=9.5cm | θ=45°; φ= 5-20mm |  | High pressure and air entrainment at the downstream of conduit facilitated aeration efficiency |
| 5 | Tuna et al. (2014) | L=2 to 6m | θ=45° | 2 and 49 | Oxygen can be transferred effectively through high head gated circular conduits. The results revealed that when Fr > 20, the increasing tendency of aeration efficiency decreased. |
| 6 | Unsal et al. (2009) | L=2m to 6m;  W=4cm;  H=25cm | θ=45°; φ=1.6cm to 4.8cm | 2.73 and 32.42 | The outcomes demonstrated the great aeration efficiency of the free-surface conduit flow systems. Nearly full oxygen transfer up to the saturation value was attained at Froude values larger than 15. |

Note Table 6; Where, L= Length of conduit; W = Width of conduit; H= Height of conduit; θ= Sluice gate lip

# Table 7: Stepped Channels

| S. No | Authors | Geometrical Characteristics | Hydraulics Parameters | Findings |
| --- | --- | --- | --- | --- |
| 1 | Khdhiri et al. (2014) | h=0.05-0.10m; l=0.10-0.14m; n=3-10W=0.15-0.3m;  H=0.25-0.5m; | Q=0.3-2.5x10^-3^m^3^/s  h_c_= 0.007-0.03 m | The maximum aeration level reached 70%. In parameter ranges, the standard error for estimating aeration efficiency was found to be less than 17%. |
| 2 | Baylar et al. (2006) | H=0.05-0.15m; W=0.35m; H=0.45m  θ = 30°,40°, and 50° | Q / unit width= 5-50m^2^/s | As step height increases, aeration efficiency generally increases for all chute angles.  In comparison to skimming flow, the nappe flow regime offers higher aeration efficiency. |
| 3 | Wan et al. (2017) | h= 0.1m; n= 9 ;l=0.25m;W=1m; θ=21.8° | Q= 0.058m^3^/s; h_c_= 0.14m | Turbulent aerated flows accelerate DO diffusion, making it easier to distribute the DO concentrations uniformly in space. |
| 4 | Baylar et al. (2009) | W=0.30m; H=0.50m;  h= 5,10,15cm; θ= 14.48°,18.74°,22.55°,30.40°,and 50° | Q= 16.67 and 166.67L/s | A nappe flow regime produces more aeration than the others |
| 5 | Cheng and Chen, (2013) |  |  | CFD model for air-water flow over stepped channel was proposed. Air inception moves downward on the surface of stepped spillways as discharge increases |
| 6 | Dermawan et al. (2017) | Two set of steps(flat and pooled steps, n=40 and 20); The stepped spillways thickness of 0.01 m and side walls with height of 0.6 m.The slopes of stepped spillway (θ) are 45˚ and 30˚ with number of steps 20 and 40, respectively  h=0.025m to 0.05 m- | Discharge per unit width (Q) = 69.13cm^2^/s to 613.38 cm^2^/s  and Froude number ranging between 1.12 and 9.91. | Increasing slope of stepped spillways is associated with higher dissolved oxygen levels, especially at Fr* < 2.00, and surface roughness of steps. The increase of dissolved oxygen from the upstream to the downstream of stepped spillway average between 30%-50%. |
| 7 | Takahashi et al. (2006) | W=1m; n =10; h=0.1m; l=0.25m; θ= 21.8° | Q = 0.017-0.04m^3^/s | When flow conditions were identical on the rough stepped chute and the smooth stepped chute, the position of self-aeration inception was consistently displaced downstream. Local air–water flow measurements also found that the rough step chutes had higher velocities than smooth steps. |
| 8 | Moulick et al.(2010) | n= 6,8,10,12, and 14; h=0.214,0.25,0.30,0.375, and 0.50m; H= 3.0m; W=0.5m; L= 0.61m | Hydraulic loading rate (q_w_) = 0.001, 0.005, 0.009, 0.013 and 0.0017m^2^/s | The aeration efficiency increases with a dimensionless vale (d_c_/h) and with number of steps. |
| 9 | Rathinakumar et al. (2015) | h= 0.15 and 0.18m; n= 12 and 10; W=0.60m; H=1.80m | Hydraulic loading rate (q_w_) = 0.005,0.001,0.0015,0.02,0.025,0.03 and 0.035 m^2^/s | Aeration efficiency increases with increase in dimensionless value (dc/h) and increase in number of steps.  It was found that aeration efficiency of recycled water was higher |
| 10 | Aras and Barkun, (2012) | n=4; W=7.5cm; H=13.5cm |  | Aeration happens with the formation of hydraulic jump and water jet. |
| 11 | Baylar et al. (2007) | h= 5,10,15cm;  W=0.30m; H=0.50m | Unit discharge ranged between  16.67 × 10^−3^ m^2^/s and 166.67×10^−3^ m^2^/s. | Aeration efficiency in stepped channels is inversely proportional to ratio of critical flow depth to step height. |

Note Table 7:- Where, h= Step height; L=Step length; n= Number of steps; W= Channel width; H= Channel height; Q= Discharge; h_c_= Critical water depth, θ= Chute angle

# Table 8: Sensitivity analysis in Stepped channel aeration with ANN Model

| Variables Combination | Parameter  eliminated | CC | MAE | RMSE |
| --- | --- | --- | --- | --- |
|  |  |  |  |  |
| E_20_= f (Q, α, N, h, l, d_c_/h ) | - | 0.9848 | 0.0259 | 0.0340 |
| E_20_= f (Q, α, N, h, l) | d_c_/h | 0.9840 | 0.0257 | 0.0324 |
| E_20_= f (Q, N, h, l, d_c_/h) | α | 0.9795 | 0.0290 | 0.0384 |
| E_20_= f (Q, α, h, l, d_c_/h) | N | 0.9238 | 0.0474 | 0.0708 |
| E_20_= f (Q, α, N, l, d_c_/h ) | h | 0.9576 | 0.0434 | 0.0572 |
| E_20_= f (Q, α, N, h, d_c_/h ) | l | 0.9704 | 0.0363 | 0.0475 |
| E_20_= f (α, N, h, l, d_c_/h) | Q | 0.8723 | 0.0697 | 0.0888 |

# Note Table 8:- Where, Q= discharge, α= chute angle, N= number of steps, h= height of steps, l= length of steps, d_c_/h = dimensionless discharge

# Table 9: Stat Indices of Stepped Channel in Training and Testing (ANN Model)

| Description | Training | Testing |
| --- | --- | --- |
| CC | 0.9891 | 0.9848 |
| MAE | 0.0217 | 0.2590 |
| RMSE | 0.0280 | 0.0340 |

Note Table 9:- refer to foot note table 4

**Table 10: Features of stepped channel dataset**

|  | *Q* | α | *N* | *h(m)* | *l(m)* | *dc/h* | *E_20_* |
| --- | --- | --- | --- | --- | --- | --- | --- |
| Mean | 67.55156 | 24.62662 | 20.56954 | 0.137715 | 3.37947 | 0.010513 | 0.635318 |
| Standard Error | 4.51128 | 1.293062 | 1.097962 | 0.008275 | 0.134109 | 0.002278 | 0.014069 |
| Median | 50 | 22.55 | 16 | 0.1 | 3.89 | 0 | 0.68 |
| Mode | 16.67 | 0 | 25 | 0.05 | 5 | 0 | 0.74 |
| Standard Deviation | 55.43554 | 15.88941 | 13.49198 | 0.10168 | 1.647956 | 0.027993 | 0.172887 |
| Sample Variance | 3073.099 | 252.4734 | 182.0335 | 0.010339 | 2.715758 | 0.000784 | 0.02989 |
| Kurtosis | -1.03189 | -0.91077 | 0.47085 | 4.013988 | 0.282098 | 7.780124 | -0.40753 |
| Skewness | 0.490694 | 0.065948 | 1.227403 | 1.958237 | -1.17847 | 2.875505 | -0.5691 |
| Range | 166.669 | 50 | 44 | 0.45 | 5 | 0.1441 | 0.742 |
| Minimum | 0.001 | 0 | 6 | 0.05 | 0 | 0 | 0.16 |
| Maximum | 166.67 | 50 | 50 | 0.5 | 5 | 0.1441 | 0.902 |
| Sum | 10200.29 | 3718.62 | 3106 | 20.795 | 510.3 | 1.5874 | 95.933 |
| Confidence Level(95.0%) | 8.913863 | 2.554968 | 2.169468 | 0.01635 | 0.264986 | 0.004501 | 0.0278 |
|  |  |  |  |  |  |  |  |

# Table 11: Fine Bubbles Diffusers

| S. No | Authors | Device used | Parameters | Findings |
| --- | --- | --- | --- | --- |
| 1 | Xu et al. (2020) | Fine bubble diffusers and impellers | Fr.:15 Hz, 17.5 Hz, 20 Hz, and 22.5 Hz, Q_A_:0.8 m3 /h, 1.2 m3 /h, 1.6 m3 /h, and 2 m3 /h, | As the air flow rate and horizontal liquid velocity increase, so does the oxygen transfer coefficient. |
| 2 | Jamnongwong et al (2016) | Fine bubble membrane diffusers and large blade slow speed mixers | B_D_(cm), B_V_(cm/s), A(1/m) | The volumetric mass transfer coefficient of liquid film forming apparatus-aeration systems is significantly higher than that of conventional systems. |
| 3 | Bunea et al. (2017) | Disperse aeration device, fitted with interchangeable perforated plate. | SOTR, SOTE, Re, A, K_La_ | A comparison of the KLa performance of various types of aerators is presented. |
| 4 | Duchene et al. (2001) | 38 wastewater plants | SAE, SOTR, SOTE | The small size of the tank implies a low volume/wetted surface ratio in comparison to large open channels, which effectively doubles the mixing power required, with a 7% impact on the SAE. |
| 5 | Fayolle et al. (2007) | fine bubble diffusers and axial slow speed mixers | A_V_, B_S_, OTC | For various operating conditions, predicted oxygen transfer coefficients are within 5% of experimental results (varying pumping flow rates of the mixers and air flow rates). The actual bubble size must be precisely known in order to estimate the oxygen transfer coefficients with optimism. |
| 6 | Zhou et al. (2013) | Two sets of identical A^2^/O bioreactors and aeration tank. | K_La_, OTR, OTE | When compared to clean water, the oxygen transfer performance of fine-bubble aerators in the aeration tank decreased dramatically. Under low-aeration conditions, the oxygen transfer coefficient was reduced by more than 50%. |

Note Table 11:- Fr.: Working frequency of impeller, Q_A_: Air flow rate, B_D_:Bubble diameter, B_V_: Bubble rising velocity, A: Interfacial area, SAE: Standard aeration efficiency, SOTR: Standard oxygen transfer rate, SOTE: Standard oxygen transfer efficiency, A_V_: Axial liquid velocity, BS: Bubble size, OTC: Oxygen transfer coefficient, K_L_a: Volumetric oxygen transfer coefficient

# Table 12: Jet Diffusers

| S. No | Authors | Geometrical Characteristics | Parameters | Findings |
| --- | --- | --- | --- | --- |
| 1 | Singh et al. (2011) | Circular, square, rectangular and rectangular with rounded edge | K_L_a_(20)_, OTE, A_f_, A_s_ , Q(2.5x10^-3^, 2.0x10^-3^,  1.6x10^-3^,  1.0x10^-3^),  P/V, P_d_, _,_J_v_ | Rectangular with rounded edge plunging jets provided the highest OTE (1.45 times), square (1.68 times), and rectangular (1.36 times) plunging jets at a given kinetic jet power. |
| 2 | Deswal and Verma, (2007) | - | K_L_a_(20)_, OTE, n, A_f,_ | K_L_a_(20)_ and OTE of m  ultiple plunging jets for air/water systems outperformed a single plunging jet significantly. |
| 3 | Shukla and Goel, (2018) | Rectangular with round edges | K_L_a_(20)_, OTE, J_n_, Q, A_s_ | The maximum OTE of 21.53 kgO_2_/kW-hr was obtained for a single nozzle aerator with a discharge of 1.11 l/s; |
| 4 | Deswal and Verma (2007) | Conical shaped plunging hollow jet | K_L_a_(20)_, OTE, Q, v_j_, t_j_ | The results indicate that the conical plunging jet aerator's K_L_a_(20)_ and OTE, are competitive with other types of aeration systems. The OTE ranged between 2.56 – 10.73 kgO_2_/kW-hr |
| 5 | Deswal (2009) | Single hollow plunging jet | K_L_a_(20)_, OTE, Q, J_t_ | K_L_a_(20)_ and OTE increases with discharge. The OTE ranged between 1.91–10.04 kgO_2_/kW-hr |

Note Table 12:- K_L_a: Volumetric oxygen transfer, OTE: Oxygen transfer efficiency, A_f_,: Flow area, A_s_ : Jet surface are per unit length, Q: Discharge, P/V: Jet power per unit volume, P_d_: Penetration depth, v_j_ : Jet velocity, J_n_ or n : Number of jets, t_j_:_:_ jet thickness

# Table 13: Sensitivity Analysis of Jet Diffusers in terms of OTE

| Variables Combination | Parameter  eliminated | CC | MAE | RMSE |
| --- | --- | --- | --- | --- |
| OTE : f (Q,V,P/V) | - | 0.9143 | 2.7069 | 4.5634 |
| OTE : f (V,P/V) | Q | 0.9073 | 2.7621 | 4.6990 |
| OTE : f (Q, P/V) | V | 0.7723 | 3.5555 | 6.1114 |
| OTE : f (Q,V) | P/V | 0.9129 | 2.7543 | 4.6025 |

# Table 14: Stat Indices of Jet Diffusers in Training and Testing in terms of OTE

| Description | Training | Testing |
| --- | --- | --- |
| CC | 0.925 | 0.9143 |
| MAE | 0.5955 | 2.7069 |
| RMSE | 0.7173 | 4.5634 |

**Table 15: Features of Jet diffusers dataset**

|  | *Discharge (l/s)* | *Velocity (m/s)* | *Power ÷ Vol* | *OTE* |
| --- | --- | --- | --- | --- |
|  |  |  |  |  |
|  |  |  |  |  |
| Mean | 2.938 | 8.437 | 0.315 | 4.24 |
| Standard Error | 0.201399 | 0.839394 | 0.068733 | 0.687702 |
| Median | 2.96 | 7.17 | 0.13 | 3.09 |
| Mode | 1.11 | 1.87 | 0.02 | 3.15 |
| Standard Deviation | 1.273757 | 5.308793 | 0.434706 | 4.349407 |
| Sample Variance | 1.622457 | 28.18328 | 0.188969 | 18.91734 |
| Kurtosis | -1.27167 | -0.49912 | 1.998905 | 7.224343 |
| Skewness | -0.06367 | 0.785915 | 1.749628 | 2.657357 |
| Range | 3.58 | 17.18 | 1.42 | 20.48 |
| Minimum | 1.11 | 1.87 | 0 | 1.05 |
| Maximum | 4.69 | 19.05 | 1.42 | 21.53 |
| Sum | 117.52 | 337.48 | 12.6 | 169.6 |
| Count | 40 | 40 | 40 | 40 |
| Confidence Level(95.0%) | 0.407367 | 1.697834 | 0.139026 | 1.391008 |
